# Inter-limb asymmetries and kicking limb preference in English premier league soccer players

**DOI:** 10.3389/fspor.2022.982796

**Published:** 2022-08-17

**Authors:** Gareth Nicholson, Tim Bennett, Aaron Thomas, Lysander Pollitt, Mike Hopkinson, Rubén Crespo, Tom Robinson, Rob J. Price

**Affiliations:** ^1^Carnegie School of Sport, Leeds Beckett University, Leeds, United Kingdom; ^2^Leeds United Football Club, Leeds, United Kingdom

**Keywords:** jumping, monitoring, football, H:Q ratio, gait

## Abstract

The aims of this study were: (1) to quantify interlimb asymmetries in EPL soccer players in the context of kicking limb preference and (2) to establish the relationship between interlimb asymmetries and measures of physical performance. Twenty-two players (age: 21.8 ± 4.4 years) from an EPL club performed a running gait assessment (20 km/h) and unilateral countermovement jumps, a CoD assessment (modified 505 test), and an isokinetic knee extension/flexion protocol using each leg. Asymmetries were quantified using the percentage difference method and Pearson's correlations were used to quantify the association between variables. Players displayed the greatest level of asymmetry in isokinetic strength measures (5.9–12.7%) and lower levels of asymmetry in gait (1.6–7.7%), jump (0.9–7.0%) and CoD (1.9–3.5%) assessments. The influence of the preferred kicking limb was most evident in the isokinetic assessment with the players showing dominance in the preferred limb for knee flexor strength and in the non-preferred limb for knee extensor strength. These manifested in the asymmetry values calculated for the hamstring:quadricep (H:Q) ratios at 60°/s (8.80 ± 7.82%) and 240°/s (11.22 ± 7.04%) and in the functional H:Q ratio (12.67 ± 8.25%). The asymmetry values for peak extensor moment at 240°/s showed a significant correlation (ρ = −0.55, *p* = 0.034) with 10 m time in the CoD assessment. These findings provide benchmark asymmetry data for soccer practitioners and reveal that kicking limb preferences may bring about interlimb differences in the H:Q ratio which raises important considerations in the design of testing batteries and injury reduction interventions.

## Introduction

Interlimb asymmetry has been defined as the difference in performance and function of one limb with respect to another (Bishop et al., 2016) and can be categorized into: anatomical, morphological, flexibility, strength, skill or outcome (Dos'Santos et al., 2021). There is an abundance of literature that has sought to characterize interlimb asymmetries in different sporting populations with a view to understanding potential implications for performance and injury occurrence. Whilst it is likely that all athletes will display some level of asymmetry (Read et al., 2018), there are a number of movements performed in soccer that are typically performed unilaterally such as kicking, jumping and changing direction). In addition, soccer players may favor particular limbs for certain tasks (Turner and Stewart, 2014) and are subject to the unique demands of different playing positions (Kovács et al., 2021). As such, the unique unilateral loading demands of soccer have likely driven widespread research into interlimb asymmetries in soccer players in recent years.

Research in soccer has sought to examine the influence of chronological age (Raya-González et al., 2020; Bishop et al., 2021a; Pardos-Mainer et al., 2021), sex (Pardos-Mainer et al., 2021), playing competition (Baroni et al., 2020) and seasonal variation (Bishop et al., 2022a,b,c) on the magnitude of asymmetry in soccer players. Whilst research has reported the asymmetry characteristics of academy-level English players, there is considerably less information relating to the interlimb asymmetry of senior English Premier League (EPL) players. Given that the EPL is one of the most physically intense leagues in Europe (Bradley et al., 2009; Di Salvo et al., 2009; Bush et al., 2017) this necessitates unique and repetitive loading demands and subsequently, multifactorial preparation strategies to optimize performance and reduce injury. Research which measures the asymmetry profiles of EPL players will therefore provide important information on the prevalence of interlimb asymmetries in elite performers and provide benchmark data for the development of monitoring, training and return-to-play strategies.

Previous asymmetry research using English players has revealed that interlimb asymmetries are an inherent characteristic when measured using a variety of testing protocols (Gonzalo-Skok et al., 2019; Bishop et al., 2021b, 2022a). Unlike investigations that show great variation in the methods used to calculate asymmetry (Bishop et al., 2016), recent research has consistently adopted the percentage difference method (Bishop et al., 2020) which facilitates the comparison of asymmetry magnitude across different investigations and populations. Importantly, the polarity of an asymmetry score calculated via this method allows the direction of asymmetry to be quantified and its use has shown that the ‘dominant limb’ rarely remains consistent for different asymmetry assessments and may change as a consequence of training or injury (Bishop et al., 2016; Dos'Santos et al., 2021). To ensure consistency in the comparisons being made over repeated time points, previous asymmetry studies in soccer have preferred to compare the left and right limbs. Whilst this approach is logical for the practitioner, it is common for soccer players to display kicking limb preferences (Rahnama et al., 2005). Uneven loading brought about by kicking limb preferences may result in the manifestation of interlimb asymmetries which may impact on the performance of certain tasks or increase the risk of injury (Pardos-Mainer et al., 2021). It is therefore surprising that very few studies (Rahnama et al., 2005; Ruas et al., 2015) have sought to account for the role of kicking limb preference when interpreting asymmetries in soccer players. Furthermore, studies that have considered the role of kicking limb preference have examined a narrow range of asymmetry measures (i.e. strength, flexibility) which do not reflect the broad range of activities (e.g. jumping, running) in soccer match play. Research which considers the context of kicking limb preference across a range of soccer-specific activities is needed to provide a more comprehensive profile of the modern-day soccer player and a better understanding of the characteristics that may result from the uneven loading experienced during kicking. For the practitioner, such information will help to inform the design and interpretation of future testing batteries and inform the design of soccer-specific training interventions.

Asymmetry research in soccer has shown contrasting findings when investigating the association between asymmetry magnitude and physical performance in soccer (Bishop et al., 2018; Raya-González et al., 2020; Pardos-Mainer et al., 2021). Consequently, it remains inconclusive whether the prevalence of interlimb asymmetries is a characteristic that practitioners should be concerned with. The contrasting findings regarding asymmetry and its implications for performance have resulted in the consensus that asymmetry is both task- and individual-specific (Raya-González et al., 2020; Pardos-Mainer et al., 2021). As a result, it has recently been recommended (Bishop et al., 2018) that multiple physical testing protocols should be used to assess the relationship between interlimb asymmetry and performance in soccer because of the multiplanar demands of the sport. Recent studies have used a combination of vertical jump, sprint, and CoD tests to quantify interlimb asymmetry in soccer players (Bishop et al., 2021a,b; Pardos-Mainer et al., 2021) but these studies have not examined other soccer-related activities such as linear running and kicking which are needed to further our understanding of soccer's unique asymmetry profiles.

A plethora of information exists on the running requirements (i.e. distances, velocities) of EPL players but very limited information exists on player's running styles and interlimb asymmetries despite similar research in other sporting populations (Tucker and Hanley, 2020; Bissas et al., 2022). In terms of measurements which relate to kicking, it is not surprising that there exists an abundance of information on the knee extensor and flexor strength given the reported links with hamstring strain injuries (Lee et al., 2018). However, there is a lack of information on the knee extensor and flexor strength of players who play in the EPL (Baroni et al., 2020) and crucially there is a failure to account for interlimb asymmetries in these characteristics; although this has been reported in other national soccer leagues (Ruas et al., 2015). Given the unique requirements of EPL match play, such information is important for practitioners for informing regular monitoring procedures, training interventions and return-to-player decisions. As such, an investigation which adopts a comprehensive asymmetry testing battery and accounts for the possible influence of kicking limb preference will provide important information that will inform monitoring, training and injury prevention practices in elite English soccer players. Therefore, aims of this study were twofold: (1) to quantify interlimb asymmetries in EPL soccer players within the context of kicking limb preference and (2) to establish the relationship between interlimb asymmetries and measures of physical performance.

## Methods

### Participants

Twenty-two male professional soccer players (age: 21.8 ± 4.4 years, body mass: 75.4 ± 7.2 kg, height: 1.81 ± 0.07 m) completed one testing session during pre-season of the 2021–22 English domestic season. All players were over 16 years of age, free from injury and played for the same football club in the EPL as part of the first-team squad. Twelve players had played full senior international for their nation and all players were outfield players (i.e., goalkeepers were not included). Twelve players identified their right leg as their preferred kicking limb with the remaining players favoring their left leg. All players were aware of the research nature of the project, with all procedures clearly explained and written informed consent obtained. The study was approved by the Carnegie School of Sport Research Ethics Committee in accordance with the Declaration of Helsinki.

### Design

To quantify interlimb asymmetries and determine associations with athletic performance, a cross-sectional design was used in which participants completed running gait, strength, jumping and CoD assessments in a randomized order. Before the commencement of testing, players were taken through a 10-min standardized warm-up by the same member of club staff which included jogging, dynamic movements and sub-maximal jumps or sprints. Although all players had regular experience of the assessments, each test was fully explained and demonstrated, and participants completed familiarization trials for each assessment to limit possible learning effects.

### Protocol

#### Running gait

After a 6-min warm-up and familiarization period, each participant ran on a gaitway-3D instrumented treadmill (h/p/Cosmos, Traunstein, Germany) at 20 km/h for 3 min. The 3-min period included the time taken to gradually reach the target speed and slow down. The treadmill incorporated four in-dwelling three-dimensional strain gauge sensors that recorded ground reaction force (GRF) data (1000 Hz) from both feet as well as temporal data. The treadmill also recorded the center of pressure position from which step length was measured. Data were collected for 30 s (beginning after 2 min), which allowed for the collection of between 43 and 50 steps per foot.

The GRF data were analyzed using the associated software (h/p/Cosmos, Traunstein, Germany) and smoothed using an eighth-order, low-pass Bessel filter (30 Hz cut-off frequency). Kinetic and spatiotemporal variables (i.e., step length, peak vertical force, ground contact time and flight time, loading rate and push-off rate) were extracted in line with previous asymmetry analyses in treadmill running (Tucker and Hanley, 2020).

#### Single-leg jumps

Following two warm-up trials, each participant performed two maximal unilateral countermovement jumps on each leg. Limb involvement was randomized and a 1-min rest period occurred between each maximal exertion. Jump trials were performed on a force platform (Kistler 9287CA; Winterthur, Switzerland) (1000 Hz) and involved a preparatory downward movement following an upright starting position (hands on hips) (Nicholson et al., 2016).

Variables to characterize jump performance were obtained using ForceDecks 2.0.7954 software (ForceDecks, London, United Kingdom) which has previously been used to provide standardized GRF analysis in countermovement jumps (Heishman et al., 2020). The software used a 20 N offset from the measured body weight to define the start of the downward phase with the start of the upward phase defined as the point of minimum vertical displacement. Jump height was calculated from take-off velocity (take-off velocity^2^/2g), whilst flight time was quantified by taking the time between contact phases during which a vertical GRF threshold of 30 N was not exceeded no vertical GRF was recorded. To quantify propulsive kinetics, peak vertical velocity of the center of mass and mean vertical power was obtained for the upward phase. To capture limb loading during the downward phase, the mean vertical force was obtained for the downward phase prior to take-off. The best out of the two trials performed by each limb was reported and selected for further analysis.

#### Isokinetic strength

Participants underwent maximal isokinetic strength assessments of the knee extensors and flexors on the right and left limbs using an isokinetic dynamometer (System 4 Pro, Biodex Medical Systems: NY, USA). Participants were seated and positioned with the hip joint placed at approximately 90° (Walker et al., 2021). The shank was attached to the dynamometer lever arm above the medial malleolus and the center of the knee joint was aligned with the dynamometer's axis of rotation (Nicholson et al., 2016). Participants completed a knee extension/flexion protocol (concentric-concentric) at 60°/s and then at 240°/s through their full range of motion. These velocities were selected to provide slow and fast testing conditions in line with previous investigations in soccer players (Baroni et al., 2020). Testing at each velocity involved three familiarization repetitions, followed by three maximal efforts in which participants were urged to extend and flex their knee “as hard and fast as possible” (Maffiuletti et al., 2016). For each angular velocity, knee extensor and knee flexor measurements were taken within the same trials and each trial was separated by 2-min of rest. After the concentric testing protocols, participants completed an eccentric knee extension protocol at 30°/s.

For each testing condition, the peak extensor/flexor moment were extracted from within the defined isokinetic range and only the best trial for each direction (extension/flexion) was analyzed. Using the peak moment data, the conventional hamstring:quadricep (H:Q) ratio (peak concentric flexor moment / peak concentric extensor moment x 100) was calculated at 60°/s and 240°/s. Furthermore, the functional H:Q ratio (peak eccentric flexor moment at 30°/s / peak concentric extensor moment was calculated at 240°/s) in line with previous investigations involving soccer players (Ruas et al., 2015).

#### Change of direction performance

Given the multi-directional nature of soccer (Maloney et al., 2017), a modified 505 test was used to measure CoD performance. Testing took place on an indoor Mondo running surface with players wearing running trainers provided by the club. Participants started from a standing position with the front foot placed 0.5 m behind the start line. Participants then performed a linear sprint of 15 m before performing a 180° turn and sprinting back to the starting line (Emmonds et al., 2019). When running back to the starting position, participants were instructed to maintain eye contact with a researcher positioned beyond the turn point and thus rotation of the torso was permitted to facilitate this. This decision was taken to simulate gameplay conditions in which players are required to run whilst maintaining eye contact with the ball or opposition player. To assess differences between limbs, participants completed trials turning off their left and right foot, with two maximal trials being performed per foot separated by a 2–3 min rest period. A pair of electronic timing gates (Witty timing system, Microgate, Bolzano, Italy) were positioned at the start (0 m) and at 10 m, this enabled times for the 0–10 m, 10–20 m and 0–20 m sections to be recorded to the nearest 0.01 s with the quickest time of the two trials used for further analysis. A force platform (Kistler 9287CA; Winterthur, Switzerland) (1000 Hz) was positioned at the 15 m turning point to measure GRF data for the final foot contact. Trials were considered valid when the final foot contact was positioned in the central area of the force platform and required the participant's foot to cross the 15 m mark. The GRF data were analyzed using Bioware v5.3.0.7 software (Kistler, Winterthur, Switzerland) to obtain contact time, vertical impulse and anteroposterior impulse in the final foot contact.

### Statistics

Descriptive statistics [mean ± standard deviation (SD)] were initially calculated for all dependent variables in Microsoft Excel before further statistical analysis was undertaken using SPSS Statistics 27 (IBM SPSS, Inc., Chicago, IL). The distribution of the data was assessed using a Shapiro-Wilk test. Within-session reliability of test measures was quantified using the intraclass correlation coefficient (ICC; 3,1) and 95% confidence intervals alongside the mean within-participant coefficient of variation (CV: [*SD*/mean] X 100). ICC's were categorized as excellent (0.90–1.00), good (0.75–0.9), moderate (0.50–0.75) or poor (<0.50) (Koo and Li, 2016). Interlimb asymmetries were computed using the percentage difference method: 100/ (maximum value) X (minimum value) X 1 + 100, as recommended for use with unilateral tests (Bishop et al., 2018). An ‘IF function’ was added to the end of the formula in Microsoft Excel which converted the asymmetry value to become negative if the non-preferred kicking limb was larger. Spearman's rank-order correlations (ρ) were used to identify relationships between the asymmetry score (percentages) for each test and the split times (raw values) in the CoD assessment. In addition, Pearson's *r* correlations were performed to assess the association between the values (raw values) in each test and the split times (raw values) in the CoD assessment. Correlations were interpreted as small (0.10–0.29), moderate (0.30–0.49), large (0.50–0.69), very large (0.70–0.89) or extremely large (≥0.90) (Hopkins et al., 2009). An alpha level of 5% was set for all tests.

## Results

**Figure 1:**
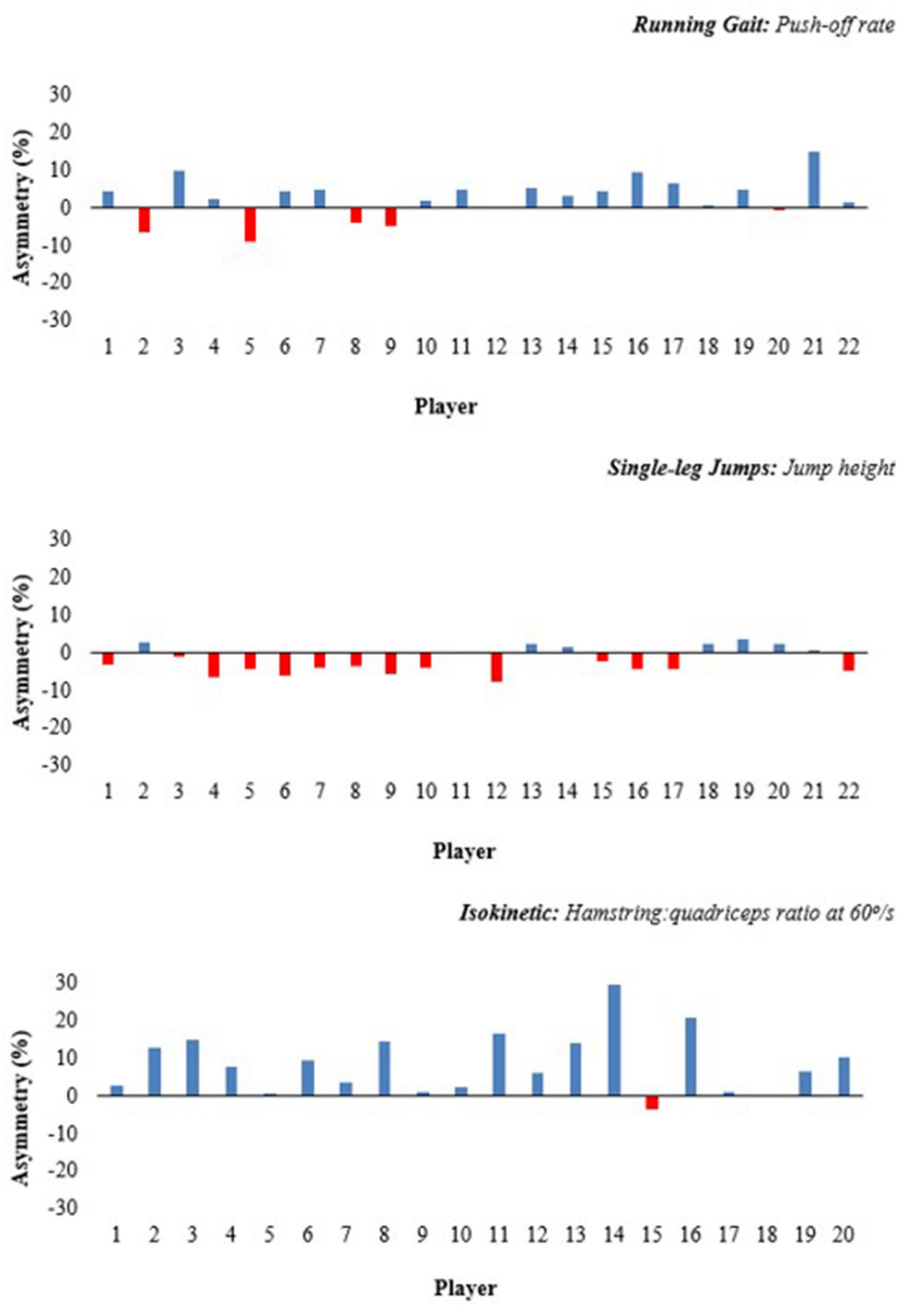
Individual asymmetry data for the top: running gait push-off rate, middle: single-leg jump height, bottom: conventional hamstring: quadriceps (H:Q) ratio measured at 60°/s. Above the line indicates a score that is greater on the preferred kicking limb and a negative score represents a value that is greater on the non-preferred kicking limb.

The Shapiro-Wilk test showed asymmetry values not to be normally distributed (*p* ≤ 0.05), but all other data were normally distributed. Table 1 displays the within-session reliability data for each test most variables showing good-excellent reliability apart from push-off rate (ICC = 0.72), 505 contact time (ICC = 0.51), vertical impulse (ICC = 0.54) and anteroposterior impulse (ICC = 0.58) which showed moderate ICC's and CV's greater than 10%. Table 2 shows the asymmetry values for each test and variable along with the percentage distribution of participants favoring the preferred or non-preferred kicking leg. There was variation in the role that the preferred kicking limb had on asymmetry values for the gait, modified 505 and jump tests but there was a larger percentage of participants favoring the non-preferred kicking limb for isokinetic peak extensor moment, and a larger number favored the preferred kicking for isokinetic knee flexor moment and H:Q ratio (Figure 1).

**Table 1:** Mean ± *SDs* for the preferred and non-preferred kicking limb and reliability data for each test variable.

**Variable**	**Non-preferred**	**Preferred**	**CV (%)**	**ICC (95% CI)**
*Running gait (treadmill)*				
Step length (m)	1.74 ± 0.07	1.73 ± 0.07	2.92	0.88 (0.75–0.97)
Peak force (BW)	3.03 ± 0.22	3.05 ± 0.21	3.16	0.83 (0.65–0.94)
Loading rate (N/s)	110.25 ± 19.34	110.04 ± 18.66	7.40	0.75 (0.52–0.91)
Push-off rate (N/s)	44.11 ± 3.72	45.30 ± 4.42	9.26	0.72 (0.50–0.86)
Contact time (ms)	178 ± 9	176 ± 9	3.49	0.82 (0.64–0.92)
Flight time (ms)	135 ± 11	135 ± 10	4.93	0.80 (0.64–0.90)
*Modified 505*				
10 m time (s)	1.76 ± 0.04	1.76 ± 0.05	1.10	0.93 (0.82–0.97)
10–20 m time (s)	2.35 ± 0.08	2.33 ± 0.09	2.33	0.85 (0.72–0.94)
20 m time (s)	4.11 ± 0.09	4.09 ± 0.12	1.30	0.88 (0.75–0.96)
Contact time (ms)	418 ± 66	415 ± 54	15.37	0.51 (0.42–0.83)
Vertical impulse (N·s)	352.33 ± 70.01	339.07 ± 57.41	15.72	0.54 (0.44–0.85)
Anteroposterior impulse (N·s)	240.15 ± 46.33	234.70 ± 39.79	14.65	0.59 (0.49–0.87)
*Single-leg jumps*				
Jump height (m)	0.25 ± 0.03	0.24 ± 0.03	3.01	0.82 (0.57–0.95)
Flight time (ms)	453 ± 24	443 ± 32	1.51	0.91 (0.79–0.96)
Peak velocity (m/s)	2.22 ± 0.13	2.19 ± 0.14	2.26	0.88 (0.64–0.98)
Mean power (W/kg)	19.26 ± 2.57	18.96 ± 2.36	3.47	0.81 (0.56–0.93)
Eccentric mean force (BW)	1.01 ± 0.02	1.01 ± 0.02	0.32	0.96 (0.85–0.99)
*Isokinetic*				
Extensor peak moment at 60°/s (BW)	0.33 ± 0.05	0.32 ± 0.06	–	–
Flexor peak moment at 60°/s (BW)	0.17 ± 0.03	0.18 ± 0.03	–	–
H:Q conventional ratio at 60°/s (BW)	51.22 ± 6.12	56.26 ± 7.75	–	–
Extensor peak moment at 240°/s (BW)	0.21 ± 0.03	0.21 ± 0.03	–	–
Flexor peak moment at 240°/s (BW)	0.14 ± 0.03	0.14 ± 0.04	–	–
H:Q conventional ratio at 240°/s (BW)	66.99 ± 10.32	68.81 ± 10.75	–	–
Ecc. flexor peak moment at 30°/s (BW)	0.24 ± 0.05	0.26 ± 0.05	–	–
H:Q functional ratio	111.29 ± 18.58	123.99 ± 21.06	–	–

*CV, coefficient of variation; ICC, intraclass correlation coefficient; CI, confidence interval; BW, body weight*.

**Table 2:** Mean (±SD) and range of asymmetry values for all variables.

**Variable**	**Asymmetry (%)**						
	**Mean ±SD**	**Range**	**% of sample**				
			**<-5**	**−5 to 0**	**0**	**0 to +5**	**>+5**
*Gait*							
Step length (m)	1.55 ± 1.39	3.35 to −5.85	5	45	14	36	0
Peak force (BW)	2.79 ± 2.13	5.60 to −9.43	5	36	0	50	9
Loading rate (N/s)	7.72 ± 5.57	19.89 to −19.00	36	9	0	23	32
Push-off rate (N/s)	4.83 ± 3.53	14.90 to −9.19	9	18	0	50	23
Contact time (ms)	1.56 ± 1.35	3.31 to −4.76	0	59	18	23	0
Flight time (ms)	2.43 ± 2.41	10.49 to −5.30	5	41	5	41	9
*Modified 505*							
10 m time (s)	1.92 ± 1.05	4.02 to −2.95	0	48	10	43	0
10–20 m time (s)	3.48 ± 2.26	7.78 to −7.34	14	57	0	19	10
20 m time (s)	2.26 ± 1.18	3.87 to −4.93	0	52	0	48	0
Contact time (ms)	12.23 ± 7.38	24.95 to −23.80	38	19	0	10	33
Vertical impulse (N·s)	14.15 ± 8.76	29.64 to −32.53	57	0	0	10	33
Anteroposterior impulse (N·s)	12.48 ± 7.56	24.26 to −29.90	52	5	0	10	33
*Single-leg Jumps*							
Jump height (m)	3.58 ± 2.07	3.57 to −7.96	23	45	0	32	0
Flight time (ms)	0.85 ± 0.37	1.10 to −1.85	0	55	0	45	0
Peak velocity (m/s)	2.75 ± 2.02	5.86 to −7.31	14	41	0	41	5
Mean power (W/kg)	2.75 ± 2.02	5.86 to −7.31	14	41	0	41	5
Eccentric mean force (BW)	5.98 ± 4.31	9.76 to −19.11	41	9	0	27	23
*Isokinetic*							
Extensor peak moment at 60°/s	5.84 ± 5.66	10.74 to −21.91	30	40	5	15	10
Flexor peak moment at 60°/s	7.22 ± 4.46	16.49 to −9.34	5	20	0	15	60
H:Q conventional ratio at 60°/s	8.80 ± 7.82	29.48 to −3.62	0	10	0	30	60
Extensor peak moment at 240°/s	7.17 ± 4.05	15.42 to −13.11	45	30	0	5	20
Flexor peak moment at 240°/s	9.34 ± 7.81	23.83 to −24.62	30	15	0	25	30
H:Q conventional ratio at 240°/s	11.22 ± 7.04	22.86 to −22.26	30	10	0	10	50
Ecc. flexor peak moment at 30°/s	9.72 ± 6.47	25.11 to −9.23	15	10	0	15	60
H:Q functional ratio	12.67 ± 8.25	34.93 to −19.83	10	10	0	10	70

*The percentage distribution of the asymmetry scores for each variable is displayed with reference to the preferred kicking limb (positive values represent a greater score on the preferred kicking limb, negative values represent a greater score on the non-preferred kicking limb)*.

Table 3 shows Pearson's correlations between the CoD split times and variables from the gait, single-leg jump, and isokinetic assessments. A number of significant negative correlations were observed between the single-leg jump variables and the split times in the modified 505 assessment (*R*^2^ = −0.24 to –−0.44). Furthermore, a number of significant negative correlations were also shown between the isokinetic performance variables and the split times in the modified 505 assessment (*R*^2^ = −0.21 to −0.35).

**Table 3:** Pearson's correlations (*r*) between the CoD split times and the variables in the gait, single-leg jump and isokinetic assessments.

**Variable**	**Modified 505**		
	**10 m (s)**	**10–20 m (s)**	**20 m (s)**
*Gait*			
Step length (m)	−0.31	−0.45	−0.19
Peak force (BW)	0.03	0.10	0.07
Loading rate (N/s)	0.02	0.15	0.10
Push-off rate (N/s)	0.25	0.02	0.14
Contact time (ms)	−0.41	−0.11	−0.25
Flight time (ms)	−0.09	−0.02	−0.06
*Single-leg Jumps*			
Jump height (m)	−0.59^**^	−0.57^**^	−0.66^**^
Flight time (ms)	−0.59^**^	−0.58^**^	−0.66^**^
Peak velocity (m/s)	−0.64^**^	−0.49^*^	−0.63^**^
Mean power (W/kg)	−0.63^**^	−0.344	−0.52^*^
Eccentric mean force (BW)	−0.06	0.08	0.03
*Isokinetic*			
Extensor peak moment at 60°/s (BW)	−0.51^*^	−0.38	−0.48^*^
Flexor peak moment at 60°/s (BW)	−0.54^*^	−0.30	−0.44
H:Q conventional ratio at 60°/s (BW)	0.01	0.21	0.14
Extensor peak moment at 240°/s (BW)	−0.56^*^	−0.48^*^	−0.58^**^
Flexor peak moment at 240°/s (BW)	−0.46^*^	−0.52^*^	−0.55^*^
H:Q conventional ratio at 240°/s (BW)	−0.20	−0.35	−0.31
Ecc. flexor peak moment at 30°/s (BW)	−0.59^*^	−0.26	−0.43
H:Q functional ratio	−0.26	0.09	−0.04

* *Significant at p < 0.05,*

** *significant at p < 0.01*.

handful of moderate-strong associations were observed between CoD split times and the asymmetry values for the isokinetic assessment. Specifically, extensor peak moment at 240°/s showed a significant correlation with the 10 m time in the CoD assessment (ρ = −0.55, *p* < 0.05). A number of other correlations were observed between the 10 m time and the asymmetry values for flexor peak moment at 60°/s (ρ = 0.413), H:Q ratio at 60°/s and (ρ = 0.360) and 240°/s (ρ = −0.42) and eccentric flexor peak moment at 30°/s (ρ = 0.40) but these did not reach statistical significance.

## Discussion

The aims of this study were twofold: (1) to quantify interlimb asymmetries in EPL soccer players within the context of kicking limb preference and (2) to establish the relationship between interlimb asymmetries and measures of physical performance. The findings showed an inconsistent influence of kicking limb preference on the asymmetry values which depended on the test and variable under observation. The effect of the preferred kicking limb was most evident in the isokinetic assessment with the players showing dominance in the preferred limb for knee flexor strength and a dominance in the non-preferred limb for knee extensor strength. In terms of physical performance, there were moderate-large correlations observed between CoD performance and certain variables in the jump and strength assessments but the asymmetry values showed fewer associations with performance. The findings suggest that practitioners should pay special attention to the impact that kicking limb preference may have on H:Q ratio which raises important considerations in the design of testing batteries and injury reduction interventions.

This is one of few studies (Rahnama et al., 2005; Ruas et al., 2015) to describe asymmetry within the context of the preferred kicking limb to reveal potential reasons behind interlimb differences. Crucially, this information is provided for the first time in EPL players which is one of the most physically intense leagues in Europe (Bradley et al., 2009; Di Salvo et al., 2009; Bush et al., 2017) and thus, strengthens our profile of athletes playing at the top level of the English game. The present findings build on existing studies within soccer by measuring interlimb asymmetry during running, jumping, CoD and strength activities, but in doing so, the data emphasizes the need to consider the reliability of individual variables when interpreting the meaningfulness of individual asymmetry scores. The reproducibility of most variables was good-excellent (Table 1) which likely reflected the player's familiarity with the testing activities. However, variables from tests that were strategy-based (e.g., modified 505 contact time/impulse) showed inherently lower levels of reproducibility which has been observed recently elsewhere (Bishop et al., 2021c). Whilst the inclusion of more comprehensive testing batteries and measurement devices (e.g. force platforms) in elite sport settings continues to be encouraged (Bishop et al., 2021c), the present findings highlight the need for caution when interpreting asymmetry values for more strategy-based metrics.

Of the assessments in the testing battery, the isokinetic parameters showed consistently higher levels of asymmetry (5.9–12.7%) than the gait (1.6–7.7%), jump (0.9–7.0%) and more ‘global’ (time-based) CoD parameters (1.9–3.5%). The magnitude of asymmetry in this study was smaller than that observed previously in male soccer players for single-leg jumps (Bishop et al., 2021a,b, 2022a), and at the lower end of values reported previously for male soccer players (Ruas et al., 2015) for knee extensor moment (6–11%), knee flexor moment (8–16%) and eccentric knee flexor moment (9–18%). Although such comparisons should be interpreted with caution because of variations in testing protocol, age and playing standard, the values reported in the present study provide up-to-date asymmetry profiles for EPL soccer players which have not been reported previously. Such information provides important benchmark information for soccer practitioners that may inform talent identification, monitoring and return-to-play decisions following injury.

The positive and negative asymmetry values seen in the present study revealed inconsistency regarding the influence of the preferred kicking limb on the interlimb asymmetries observed (Table 2 and Figure 1). The inconsistent nature of asymmetry has been previously reported (Pardos-Mainer et al., 2021) and is in agreement with the suggestion that it is both task- and individual-specific (Raya-González et al., 2020). Despite the inconsistency in the running, jumping and CoD data, there was a strong influence of kicking limb preference on the agonist-antagonist muscle strength ratio around the knee. Over 70% of players displayed greater scores for peak knee extensor moment for the non-kicking limb and 75% of players demonstrated greater scores for peak concentric (60°/s) and eccentric flexor moment in the preferred kicking limb which resulted in consistently higher conventional (90% of players) and functional (80% of players) H:Q ratios on the preferred limb. The fact that the influence of the kicking limb on asymmetry scores was most pronounced in the isokinetic strength test is not surprising and this likely reflects the obvious mechanical similarities between the soccer kicking and the isokinetic testing protocol. In terms of the influence on H:Q ratios, the knee flexor dominance of the preferred kicking limb is consistent with the observations of Ruas et al. (2015) in Brazilian soccer players and likely reflects the need for antagonist strength to decelerate repetitive kicks in the preferred kicking limb. For the non-preferred kicking limb, the higher knee extensor strength likely reflects the supportive strength required for kicking and unilateral jumping, but also the reduced need for the knee flexors in this limb to be used as a decelerator. Considering the prevalence of hamstring strains in soccer players and the reported links between knee extensor/flexor strength and hamstring strain injuries (Lee et al., 2018), practitioners should pay special attention to the influence of kicking limb preference on the H:Q ratio when designing injury-reduction strategies.

In terms of physical performance, the CoD split times showed moderate-strong associations with the jump and isokinetic measures (raw values) but the same associations were not observed with the running gait variables. The lack of association between the CoD and running variables likely reflects the multi-directional CoD component of the modified 505 test and the fact that the running protocol was performed at submaximal running speeds. In contrast, the associations between CoD performance and the jump and isokinetic variables aligns with research which emphasizes the importance of knee extensor and flexor strength for braking and acceleration activities (Harper et al., 2021) and underlines the need for practitioners to develop this through physical conditioning programmes. When considering the specific influence of asymmetry on athletic performance, only the relationship between isokinetic extensor moment and 10 m time reached statistical significance (ρ = −0.55, *p* < 0.05). Whilst there are studies that have also reported associations between larger asymmetries and slower CoD times (Hoffman et al., 2007; Bell et al., 2014; Bishop et al., 2021b, 2022b), there are examples of studies which have shown no associations between these measures (Bishop et al., 2021a, 2022b; Pardos-Mainer et al., 2021). The correlational analyses performed in this study suggest that improvements in 10 m time may be obtained by reducing interlimb asymmetries in knee extensor strength. However, in the absence of other correlations between asymmetries and CoD performance, the present findings provide a stronger case for practitioners to prioritize the development of strength and power when seeking to optimize CoD performance. Future research which adopts a more longitudinal design is needed to better understand the performance implications that may result from interventions which reduce interlimb asymmetries.

When interpreting the findings of this study, practitioners should be mindful of some limitations. Firstly, the investigation utilizes players from one EPL team and therefore further research is required to understand whether the reported asymmetry profiles are representative of other teams with differing playing and training strategies. Whilst the use of specific measurement devices (e.g. force platforms) permitted the investigation of more in-depth metrics, the use of an indoor running track and treadmill protocol limited the ecological validity of some assessments for a soccer population. Finally, the inclusion of other assessments related to soccer match play (e.g. drop jumps, sprint performance) would add to the asymmetry profile of EPL players and enable further insights into the links between interlimb asymmetry and physical performance.

## Conclusions

EPL soccer players display the greatest level of interlimb asymmetries in isokinetic strength measures and lower levels of asymmetry in running gait, jump and CoD assessments. There was an inconsistent influence of the preferred kicking limb on interlimb asymmetries for running gait, jump and CoD assessments. In contrast, EPL players showed a dominance in the preferred limb for knee flexor strength but a dominance in the non-preferred limb for knee extensor strength. These findings highlight that kicking limb preference may therefore bring about interlimb differences in the H:Q ratio which raises important considerations in the design of testing batteries and injury reduction interventions. In terms of physical performance, the CoD test showed few correlations with the interlimb asymmetry values but stronger relationships were observed with the raw strength and jump data. As such, the present data indicates that practitioners should prioritize the development of strength and power characteristics when seeking to optimize CoD performance.

## Data availability statement

The raw data supporting the conclusions of this article will be made available by the authors, without undue reservation.

## Ethics statement

The studies involving human participants were reviewed and approved by Leeds Beckett University Ethics Committee. The patients/participants provided their written informed consent to participate in this study.

## Author contributions

RC, TR, and RP arranged data collection within the football club. GN, TB, AT, LP, and MH performed data collection. GN processed the data and created the figures. All authors conceptualized and designed the study, wrote the manuscript, interpreted the results of the research, edited, critically revised, and approved the final version for submission.

## Funding

The data collection was supported by funding provided by Leeds United Football Club as part of a wider sport science support project; however, the nature of the data is purely descriptive and not associated with any governing body, commercial sector, or product. No funding was provided for the writing of this manuscript. The results of the present study do not constitute endorsement by Leeds United Football Club.

## Conflict of interest

The authors declare that the research was conducted in the absence of any commercial or financial relationships that could be construed as a potential conflict of interest.

## Publisher's note

All claims expressed in this article are solely those of the authors and do not necessarily represent those of their affiliated organizations, or those of the publisher, the editors and the reviewers. Any product that may be evaluated in this article, or claim that may be made by its manufacturer, is not guaranteed or endorsed by the publisher.
